# Development of mobile phone-based dietary data collection applications in pregnant women and infants for the M-SAKHI trial

**DOI:** 10.1017/jns.2023.95

**Published:** 2023-12-13

**Authors:** Shilpa Bhaise, Archana Patel, Varsha Dhurde, Michelle Almeida, Tran Do, Sumithra Muthayya, Michael Dibley

**Affiliations:** 1 Lata Medical Research Foundation, Nagpur, Maharashtra, India; 2 Datta Meghe Institute of Medical Sciences, Sawangi, Maharashtra, India; 3 Sydney School of Public Health, The University of Sydney, Sydney, New South Wales, Australia; 4 National Institute of Nutrition, Hanoi, Vietnam; 5 The Sax Institute, Ultimo, New South Wales, Australia

**Keywords:** Food Frequency Questionnaires, Dietary assessment, Mobile-based dietary application, 24-h dietary recall

## Abstract

In nutritional epidemiological studies, it is imperative to collect high-quality data to ensure accurate dietary assessment. However, dietary data collection using traditional paper forms has several limitations that may compromise data quality. The aim of this study was to propose novel methods to design and develop software applications (Apps) for dietary data collection to assess the nutritional status of pregnant women and infants. This study is part of the M-SAKHI (Mobile-Solutions for Aiding Knowledge for Health Improvement) cluster randomised controlled trial (cRCT) implemented in central India. Three tablet-based software Apps were developed in this study: the ACEC (Automated Coding and Energy Calculation) App to establish a generic cooked food recipe database, the FFQ (Food Frequency Questionnaire), and the IDR (24 h Infant Dietary Recall) Apps to collect dietary data from pregnant women and their infants from rural area of Bhandara and Nagpur districts. Regional food lists, recipes, and portion resource kits were developed to support the data collection using the Apps. In conclusion, the Apps were user-friendly, required minimal prior training, had built-in validation checks for erroneous data entry and provided automated calculations. The Apps were successfully deployed in low-resource rural settings to accurately collect high-quality regional cooked food data and individual-level dietary data of pregnant women and their infants.

## Introduction

Maternal undernutrition,^(1,2)^ inappropriate breastfeeding, and inadequate complementary feeding practices result in poor fetal growth, subsequent infant growth, and may increase infant morbidity.^(3,4)^ During pregnancy, the dietary intake ought to be sufficient to meet the increased need for important nutrients. According to India's National Family Health Survey (NFHS-5) conducted from 2019 to 2021, about 52⋅2 % of pregnant women were anaemic (haemoglobin <11 g/dl).^(5)^ To address these problems, the Government of India launched *POSHAN Abhiyan, Janani Shishu Suraksha Karyakram, Pradhan Mantri Surakshit Matritva Abhiyan, Rashtriya Bal Swasth Karyakram,* and National Nutrition Anaemia Prophylaxis Programme.^(6)^ Periodic national nutrition surveys are conducted to evaluate diet and food consumption patterns in women and children. They use retrospective dietary assessment methods based on food consumption recall, such as 24 h Dietary Recall and Food Frequency Questionnaires (FFQs).^(5,7)^ However, dietary assessments at the community level are fraught with many challenges. It requires rapport building with respondents and expertise in how questions are asked to collect reliable information from caregivers regarding food consumed by the mother or her infant. Some of these challenges can be addressed by rigorous training. Still, a major challenge is the lack of a comprehensive nutrient database of home-cooked foods for different regions in India.^(8,9)^ Estimation of the nutritive value of home-cooked food is arduous and difficult. To assess the nutritional content of locally consumed Indian foods, a compromise has to be made by pooling relevant data from international databases. The Indian Food Composition Table (IFCT) contains nutrient values only of raw food ingredients.^(10)^ Therefore, analysis of nutrient values of diets consumed by Indian pregnant women and infants is difficult. Inaccurate dietary assessment may hinder reliable evaluation of presence of a nutrition deficiency, the impact of a nutrition intervention, and can misdirect a public health intervention.

The M-SAKHI (Mobile-Solutions for Aiding Knowledge for Health Improvement) was a cluster randomised controlled trial (cRCT) that the Lata Medical Research Foundation (LMRF), Nagpur, India, implemented in collaboration with the University of Sydney, Australia.^(11)^ This was a behaviour change communication intervention implemented by 150 community health workers (ASHA or Accredited Social Health Activists) in 119 village clusters in addition to the usual maternal and child health programmes to primarily reduce the rate of infant stunting by impacting maternal and infant diet. The intervention was received by 1250 women in their second trimester through pregnancy till the infant was 12 months of age, and the control clusters recruited 1251 pregnant women from 244 villages. The focus of the present study was to develop mobile-based software Apps to collect dietary data for this M-SAKHI trial. The dietary assessments of all pregnant women in their third trimester of pregnancy and in 840 random sub-samples of infants from all clusters were conducted periodically (10th, 12th, 15th and 18th months of age) using the following android tablet-based Apps, namely, the Automated Coding and Energy Calculation (ACEC) App, the FFQ App, and the 24 h Infant Dietary Recall (IDR) App. The ACEC App was deployed for efficient data collection of regional cooked food recipes. The FFQ and IDR Apps helped to quantify the frequency and consumption of cooked or raw food for conventional portion sizes used in the study region. This manuscript describes these innovative Apps-based methodologies used for dietary data collection to answer the question of finding ways to collect high-quality dietary data in a low-resource setting.

## Materials and methods

The steps (Process A) followed for developing a standardised recipe list of cooked foods and their energy calculation using the ACEC App are described in this section. We also describe the features of the FFQ and IDR Apps (Process B) that enabled the dietary data collection of the M-SAKHI trial participants.

### Process A

**Fig. 1.**
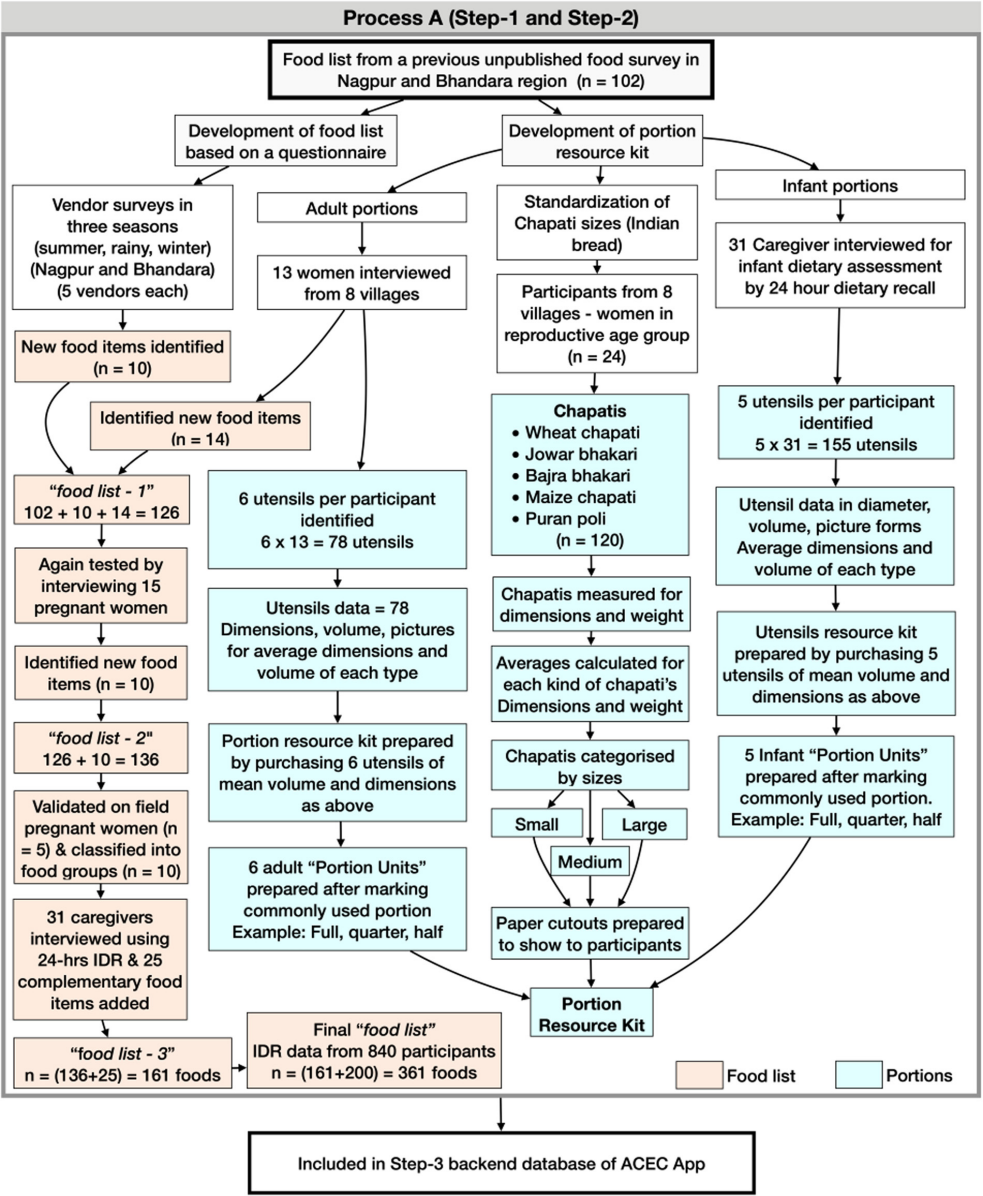
Step-1: preparation of the food list and step-2: development of *portion resource kit* of the food items.

The following backend databases: (i) location database, (ii) food ingredient database, and (iii) portion database were required to build the ACEC App for recipe data collection (Fig. 1). The location database consisted of the names of ten clusters and their fifty villages (each cluster had approximately five villages). The food ingredient database consisted of the following: *‘food list’,* IFCT for raw foods (*n* 596), non-IFCT for packaged foods (*n* 45), and the United States Department of Agriculture (USDA) for nutrient values of raw foods.^(12)^ The nutrient values were sourced from IFCT, USDA, and other published literature.^(13–15)^ The portion database consisted of the *‘portion resource kit’,* the images of the utensils and their associated weights.

#### Step 1: preparation of the ‘*food list*’

**Fig. 2.**
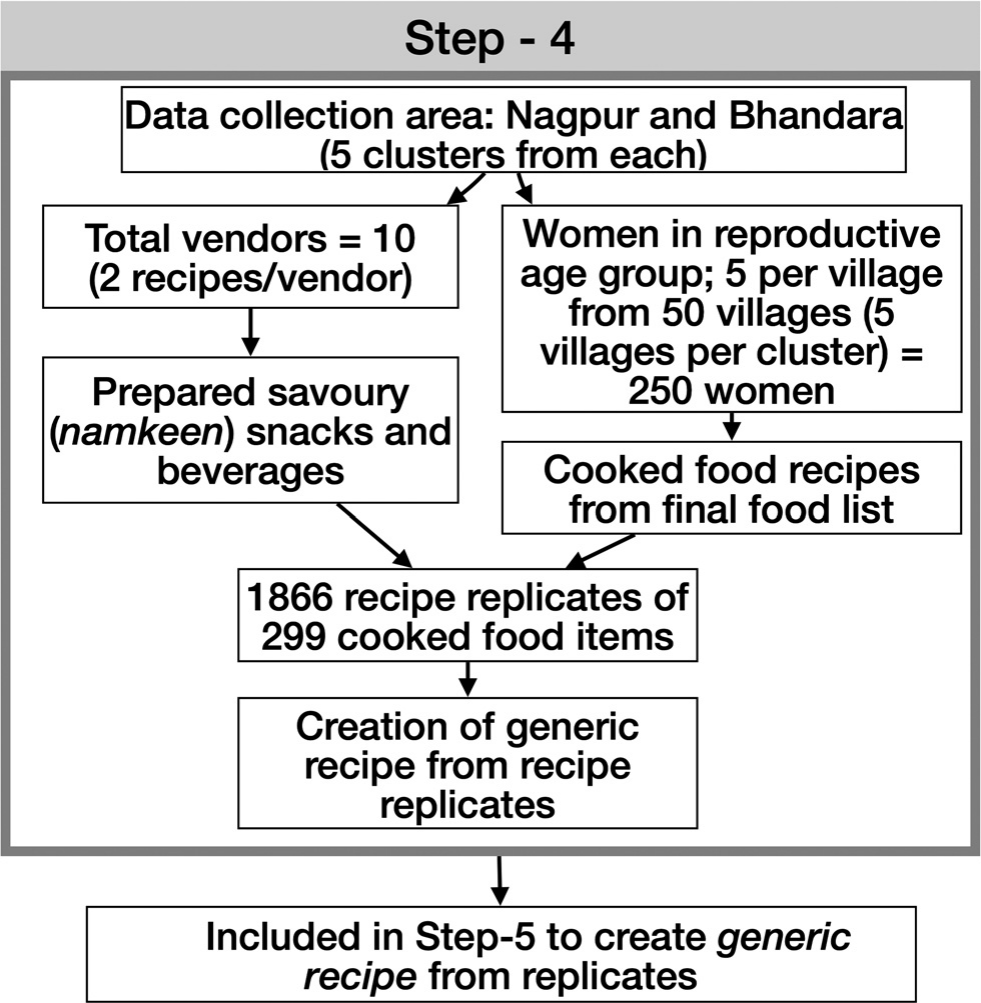
Step-4: Cooked food recipe data collection using ACEC App.

We began with a food list from an unpublished study of 102 food items prepared from 24 h dietary recalls of 111 pregnant women from the Nagpur and Bhandara districts of Maharashtra in India. This list was updated with twenty-four new food items that consisted of common foods, foods consumed during pregnancy, festival foods, special occasion foods, and seasonal foods by interviewing ten local market vendors in all three seasons (summer, rainy, and winter) and also interviewing thirteen women between 18 and 45 years of age. We labelled this updated food list of 126 (i.e. 102 + 24) food items as *‘food list-1’*. The *‘food list-1’* was further revised to *‘food list-2’* of 136 items by interviewing another fifteen pregnant women from the same area. It was then validated in nine other pregnant women. The food items in *‘food list-2’* were classified into food groups: cereals, lentils, leafy vegetables, other vegetables, non-vegetarian, savoury snacks (namkeen), sweets, beverages, condiments, and dairy. To capture an infant's commonly consumed food items, 24 h dietary recalls were also conducted in the non-study infants from selected villages from the same districts. Twenty-five complementary food items were identified, and the *‘food list-2’* was updated (136 + 25 = 161) to create a *‘food list-3’.* This *‘food list-3’* was further updated with 200 new food items consumed by infants identified during IDR data collection. Of these, sixty-two were market packaged foods that included bakery products, chocolates, and biscuits. This resulted in the final *‘food list’* with 361 food items (Fig. 2). This final food list was incorporated into the ACEC App. In each of these Apps, the English names of food items were translated into the Marathi language.

#### Step 2: development of *‘portion resource kit’* of food items

Pregnant women (*n* 13) from villages of eight different clusters were asked to bring common utensils used during meals. Six utensils (a cup, plate, bowl, teaspoon, tablespoon, and glass) per respondent were selected (seventy-eight utensils). The dimensions and volumes of the utensils were measured three times, and the average value for each type of utensil was calculated. Utensils with these average dimensions were purchased to prepare the *‘portion resource kit*’, consisting of six adult portion units: a cup, plate, bowl, teaspoon, tablespoon and glass. Each portion was marked with a graduated scale from 0 to 1 with intervals of 0–25 to indicate quarter, half, three-quarters, and full measures. The Indian bread or *chapati* is consumed in nearly every meal in the region and can be prepared from different types of cereal, i.e. wheat, millets (*bajara*, *jowar)* or maize and *puranpoli* (a commonly consumed preparation of wheat, jaggery, or sugar). We standardised the dimensions of the *chapati* by requesting twenty-four respondents from villages of the eight different clusters to prepare at least one of each type. A total of 120 *chapatis* were prepared. The diameter and weight were recorded for each of the *chapatis*. These dimensions were used to categorise *chapatis* into three sizes, i.e. small, medium, and large. Corresponding to these sizes, paper cutouts were prepared and included in the *portion resource kit*. The *portion resource kit* for infants was also created by asking the thirty-one caregivers to bring five utensils used to feed the infants (155 utensils). The dimensions and volumes of these utensils were measured by the same method used for scaling adult portion units. The standardised infant portions were infant cup, infant bowl, infant plate, infant glass, and infant spoon.

#### Step 3: development and testing of the ACEC App software

The CommCare version 2.20 (Dimagi, Inc., USA) was used to develop the ACEC App for android operating system and was installed on a Lenovo tablet.

##### Testing and piloting

The ACEC App was tested by entering data from fifteen different cooked food recipes. The testing errors, such as the App crashing and the failure of the dropdown list to appear, were identified. The resolution of errors was a back-and-forth process between the nutritionist and the developers. When all App errors were resolved, it was piloted by a nutritionist in the field for data entry of cooked food recipes from the five households.

##### Training of data collectors

The data collectors who were proficient in using android phones were recruited to use the ACEC App for cooked food recipe data collection. Nutritionists rigorously trained the data collectors for two days of in-house training, followed by on-field training. These training sessions focused on the basic knowledge of foods and food groups, practical demonstrations, accurate weighing techniques and data entry of different cooked food recipes.

#### Step 4: cooked food recipe data collection using the ACEC App

The data collection was conducted under the supervision of the nutritionists. ASHAs recruited five women from fifty villages (250 women) in ten different clusters to cook two food items each. Women aged 18–45 years who were not engaged in the Integrated Childhood Development Scheme (ICDS) of the government and who consented to prepare the cooked food items were recruited. Women engaged in the ICDS were excluded to prevent health-related biases in food preparation, e.g. using less oil. The cooked food recipes from the final ‘*food list’* were allocated to the cooks. The cooking process was undertaken at a school, Anganwadi center or at the residence of an ASHA. The recipe's main ingredients, stove and fuel, were provided to the participants, while the cooks brought their own cooking medium (e.g. oil) and spices. The *‘food list’* also contained many savoury (*namkeen*) cooked food items and juices that households obtained from vendors. For these savoury food items, we recruited ten vendors to prepare these food items, and the study nutritionist observed the cooking of the recipes. The cooks were asked to select the ingredients they usually used to prepare a cooked food item. The raw ingredient in each recipe was quantified using a standard weighing scale (Model: 1010SS, Brand: SALTER) followed by weighing the total cooked food amount for each cook. This information was used to standardise the number of ingredients in 100 g of cooked food recipe for energy calculation.

#### Step 5: method of creation of generic recipe from replicates

**Fig. 3.**
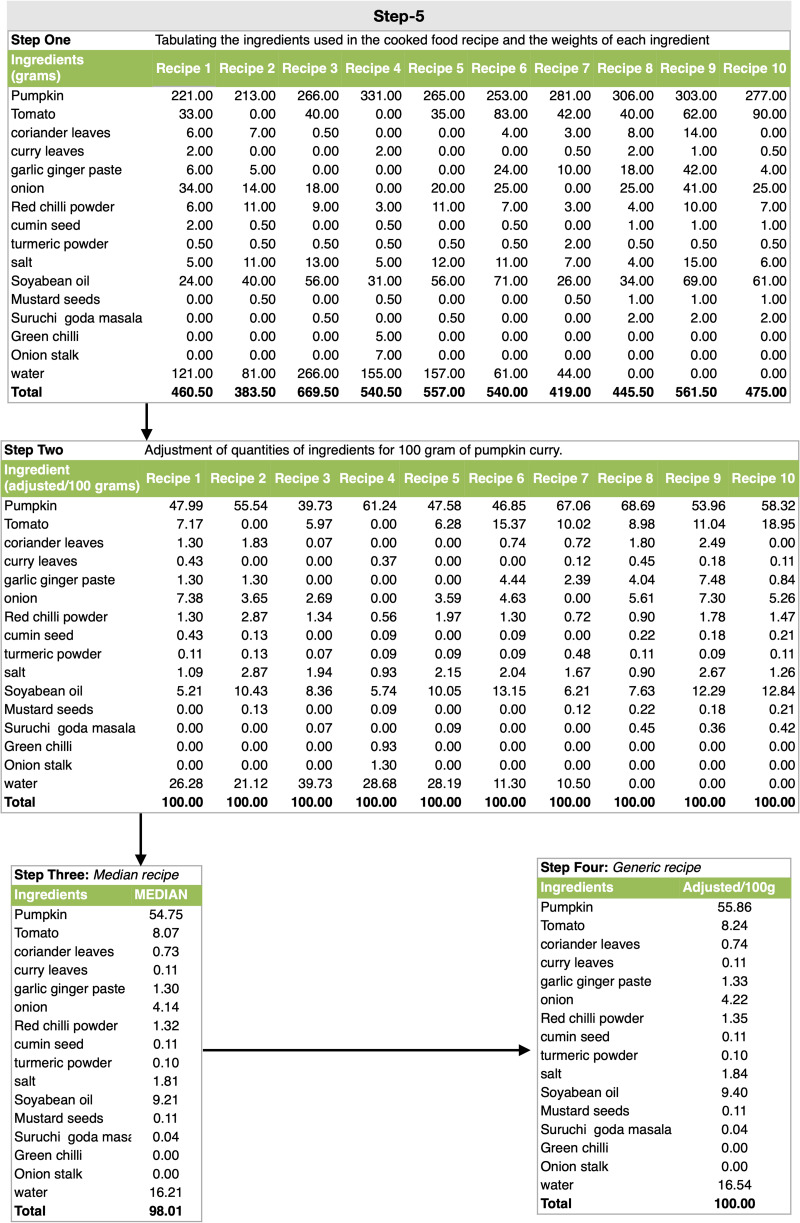
Step-5: Method of creation of generic recipe from replicates.

The cooked food recipes categorised in their food groups are shown in Fig. 3. A total of 1866 cooked food recipes were collected using the ACEC App from ten study clusters. There were multiple replicates of the same cooked food recipe. For e.g. ‘*pumpkin curry*’ could have been cooked in many different ways due to the variation in quantity and quality of ingredients used by different cooks. A ‘*generic recipe’* for every cooked food item was created from the replicates.^(16)^ This standardisation process consisted of the following steps: (i) tabulating the ingredients used in the cooked food recipe and the weights of each ingredient, (ii) the value of the ingredient quantities in the food recipe (e.g. *pumpkin curry*) were adjusted such that the total weight of cooked recipe was equal to 100 g, (iii) after this adjustment, the median weight value of each ingredient was extracted to create a ‘*median recipe*’ of that cooked food, (iv) if the total weight of the *median recipe* did not total to 100 g, then the ingredient values of this recipe were adjusted such that the total equalled to 100 g (Fig. 3). This *median recipe* with total ingredient weight of 100 g was renamed the ‘*generic recipe’*. This process yielded a total of 299 standardised regional *generic recipe*s. These recipes were then used as a recipe database for the FFQ and IDR Apps.

### Process B

#### Development of semi-quantitative FFQ App

FFQ is a type of dietary assessment method that captures the food item consumption of a respondent by inquiring about the frequency and portion or amount of food consumed in the past three months. In the App the information was collected using a repeated loop format, i.e. multiple-pass method. The FFQ App questionnaire included a *‘food list-2*’ of 136 foods (Fig. 1) as complimentary food information was obtained later; moreover, few pictures of rare vegetables that were identified by different names by different villages were also included. The FFQ queried each participant with the following three questions for every food item: (i) *Frequency of consumption*: Participants were initially asked to specify the frequency of their food consumption over the past three months. Response options included daily, weekly, monthly, or never in the last three months, (ii) *Portion size*: Participants were then asked to provide information about the portion size they typically used during consumption. Portion sizes were categorised according to measuring units such as laminated paper cutouts for Indian bread sizes (large, medium, and small), standard units like cups, plates, bowls, teaspoons, tablespoons, and glasses for curries and beverages, and a numerical count for items like biscuits, (iii) *Number of portions consumed*: Participants were further inquired about the number of portions they consumed for the chosen portion size. Briefly, the FFQ App captured the dietary data twice during the pregnancy period, firstly between 22 and 24 weeks of gestation (second trimester) and then 3 months later, between 34 and 36 weeks of gestation (third trimester). The FFQ App also included adult portion resource kit images in the input interface and their corresponding weights in the backend. In addition to the FFQ, a separate Dietary Habit Questionnaire (DHQ) was also employed to collect qualitative data from the participants. This decision to maintain a separate questionnaire was made to ensure a seamless flow of interviews and to avoid disruption during FFQ administration. The DHQ encompassed qualitative questions, including: (i) *Food habit description*: Participants were asked to describe their food habits as either vegetarian (excluding eggs, goat, chicken, or fish) or non-vegetarian, (ii) *Changes in diet*: Participants were questioned about any modifications made to their usual diet since becoming pregnant. Responses included changes such as discontinuation, initiation, or increased consumption of specific foods, (iii) *Household oil preference*: Participants were asked to identify the most commonly used cooking oil in their household, (iv) *Medicinal and food supplements*: Participants were inquired about their consumption of medicinal (IFA consumption) or food supplements such as take home rations from Anganwadi.

#### Development of the IDR App

The IDR App was build using the multiple-pass method to collect longitudinal dietary data of infants aged 10th, 12th, 15^th^, and 18th months. The 24 h period was defined as the time from when the infant woke up on the previous day until the time the infant woke up on the interview day. The IDR questionnaire included the infant's ID, illness history, breastfeeding duration, frequency of breastfeeding and foods and beverages consumed by an infant in the past 24 h. The adult and infant portion resource kit images were available on the App with their corresponding weights. Additionally, the IDR questionnaire also queries a participant for: (i) the time when the food item was consumed, (ii) the name of the food or beverage consumed, (iii) the place of feeding: home or outside, (iv) the portion size, and (v) the quantity or number of portions consumed.

Testing and piloting of FFQ App and IDR App: The Apps were tested in the field by interviewing ten participants. Testing errors such as App crashing and failure dropdown menu items were identified. The errors were resolved by the nutritionist and developers. It was then piloted by interviewing ten non-study respondents from the study area. Nutrition experts observed the data collection process as the data collectors used the Apps. These observations specific to the usability aspects of the Apps were: (i) the ease of App navigation for data collectors, encompassing actions such as one-tap interactions to access menus or progress to subsequent sections, (ii) the clarity of questions for data collectors, evaluating their ability to read and comprehend the content, (iii) the efficacy of communication between data collectors and participants during the question delivery, (iv) the functionality of App when a data collector captured images of the supplements, (v) the ease for a participant to effortlessly identify a food items displayed by the App, (vi) the ease for data collectors to locate appropriate menu items within the App after concluding the FFQ interview, for the purpose of transitioning to the DHQ interview, (vii) the recognition and responsiveness of data collectors to alerts displayed in the App, particularly concerning data entry errors followed by the ability of data collectors to revisit these questions for the purpose of making corrections were also scrutinised.

Training of data collectors: FROs (Field Research Officers) who were qualified ANM (Auxiliary Nurse Midwife) were recruited. They were trained for three days on how to use the Apps. The first two days of training focused on the concept of nutrition, technical details and demonstration of the App. On the last day, they used the App independently to enter dietary data.

### Ethical approval

The trial was approved by three Institutional Review Boards: (1) Local IRB: LMRF, Sir Gangadharrao Chitnavis Trust and (2) Indian National IRB: Indian Council of Medical Research, and (3) University of Sydney Human Research Ethics Committee (Ref: 2015/990). A written signed informed consent was requested from each study participant in the presence of a witness prior to any data collection. Also, at the time of infant dietary data collection, the infant's primary guardian, i.e. infant's mother or caregiver gave a written informed consent for the infant's participation in this study. A copy of this consent was also given to the respondent or caregiver. Trial Registration Number: CTRI/2018/02/011915.

## Results

The processes used for preparing the three Apps resulted in the development of a list of foods consumed in the region, their recipes, portion resource kits and three Apps — ACEC App, FFQ App, and the IDR App, the features of which will be describedin the following.

### Food lists

A final food list (cooked, raw, and packaged food) of 361 items resulted from a process of repeated updating of food lists. This updating started from an unpublished list of 102 food items from a previous survey in the region of women in the reproductive age group. It was first updated to ‘*food list-1*’ of 126 items, then to ‘*food list-2*’ of 136 items, then ‘*food list -3*’ of 161 food items and finally, the food list of 361 items (Fig. 1).

### Cooked food recipe data using the ACEC App

Using the final food list of 361 items in the ACEC App, 299 cooked food items were selected, as others were raw food items or packaged foods, e.g. biscuits. These resulted in 1866 recipes that were replicates of 299 recipes. From the 1866 recipes, 299 *generic recipes* with their ingredients were developed for inclusion in the FFQ and IDR Apps used for the dietary assessment.

### Portion resource kit

**Fig. 4.**
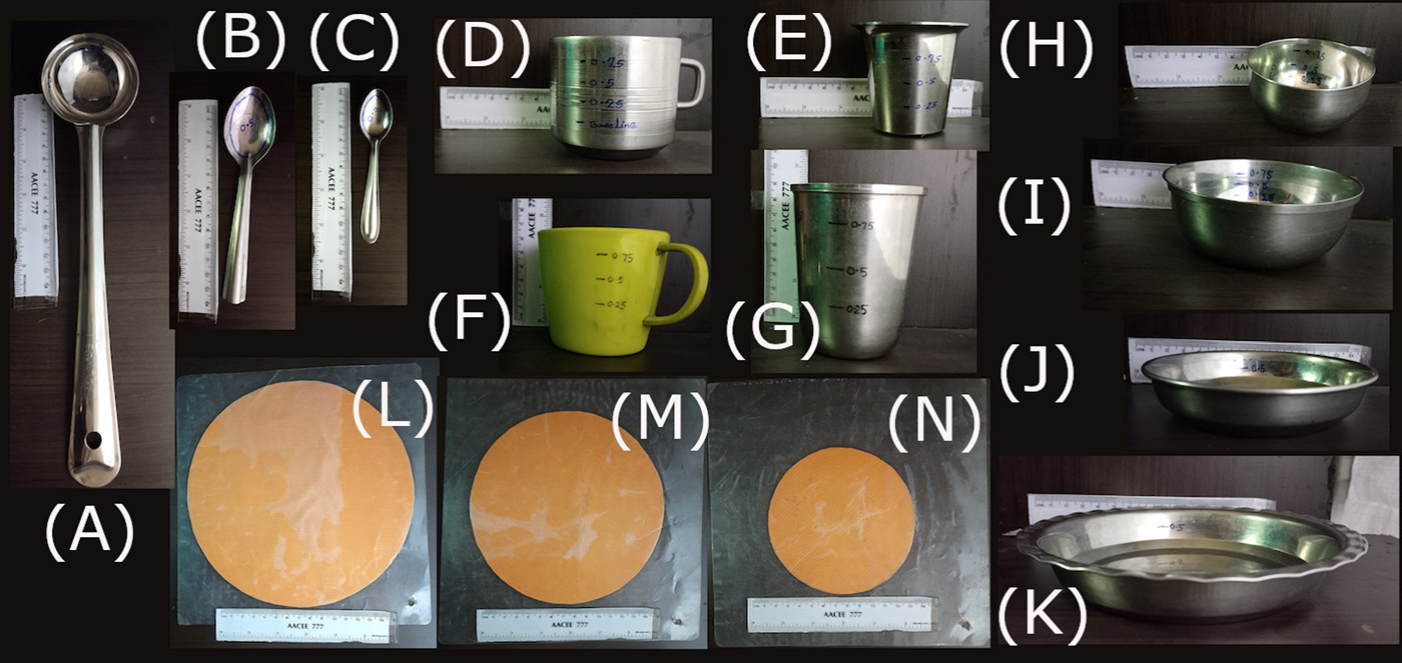
Photographs of portion sizes: (a) tablespoon, (b) teaspoon, (c) infant spoon, (d) infant cup, (e) infant glass, (f) cup, (g) glass, (h) infant bowl, (i) bowl, (j) infant plate, (k) plate; chapati cutouts sizes: (l) large, (m) medium, (n) small.

We developed two sets of resource kits: adult and infant. The adult portion resource kit consisted of common household utensils: plate, bowl, teaspoon, glass, cup, and serving spoon with a graduated scale to quantify the adult portion size of foods. The kit also consisted of laminated paper cutouts for Indian bread in three sizes: small, medium and large. The infant portion resource kit consisted of an infant plate, infant bowl, infant spoon, infant glass and infant cup that were used to quantify foods an infant consumes (Fig. 4). These portion resource kit images, and their corresponding weights were included in the ACEC, FFQ and IDR Apps.

### ACEC App features

**Fig. 5.**
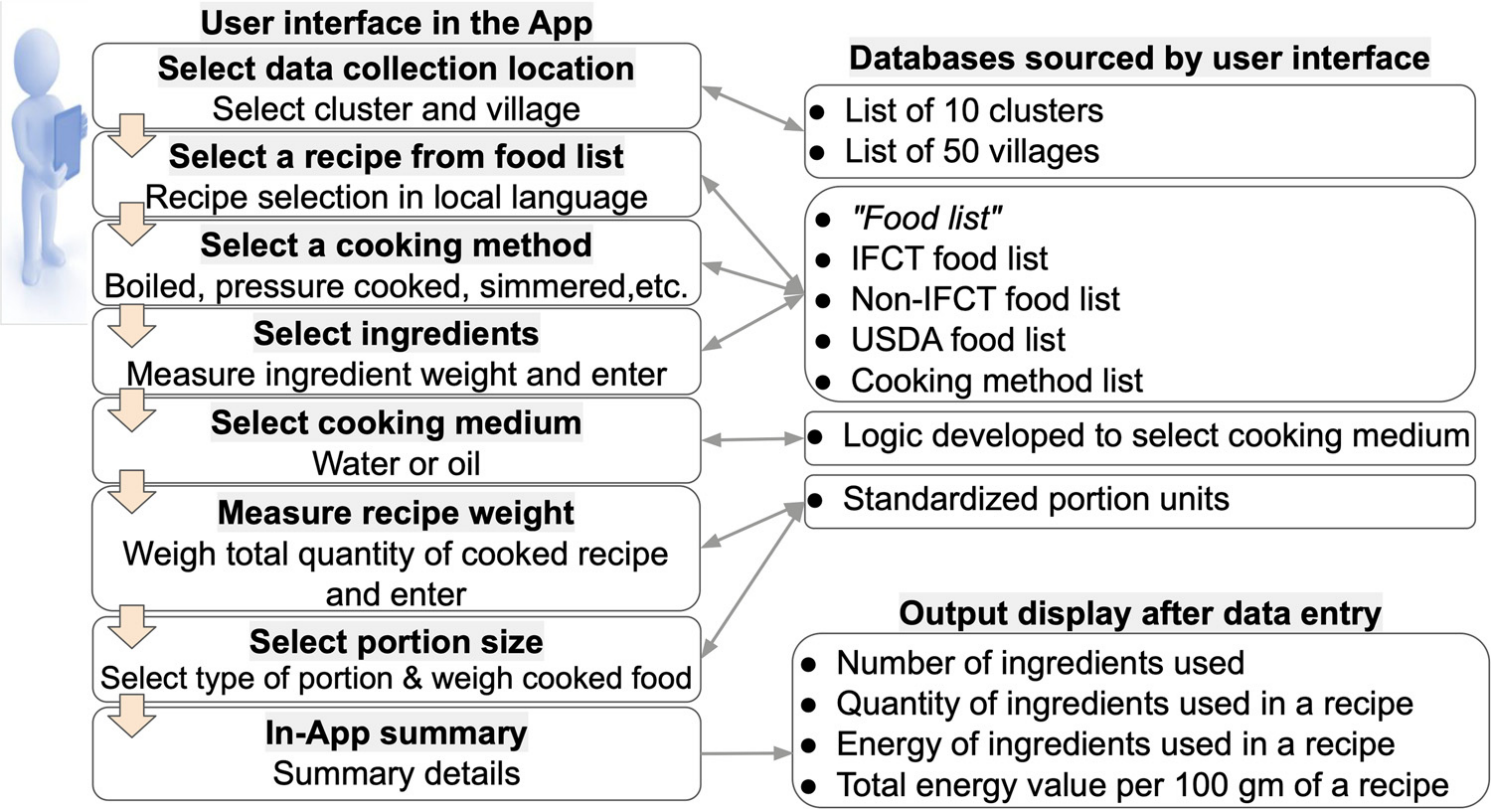
User interface and backend database of ACEC App.

**Fig. 6.**
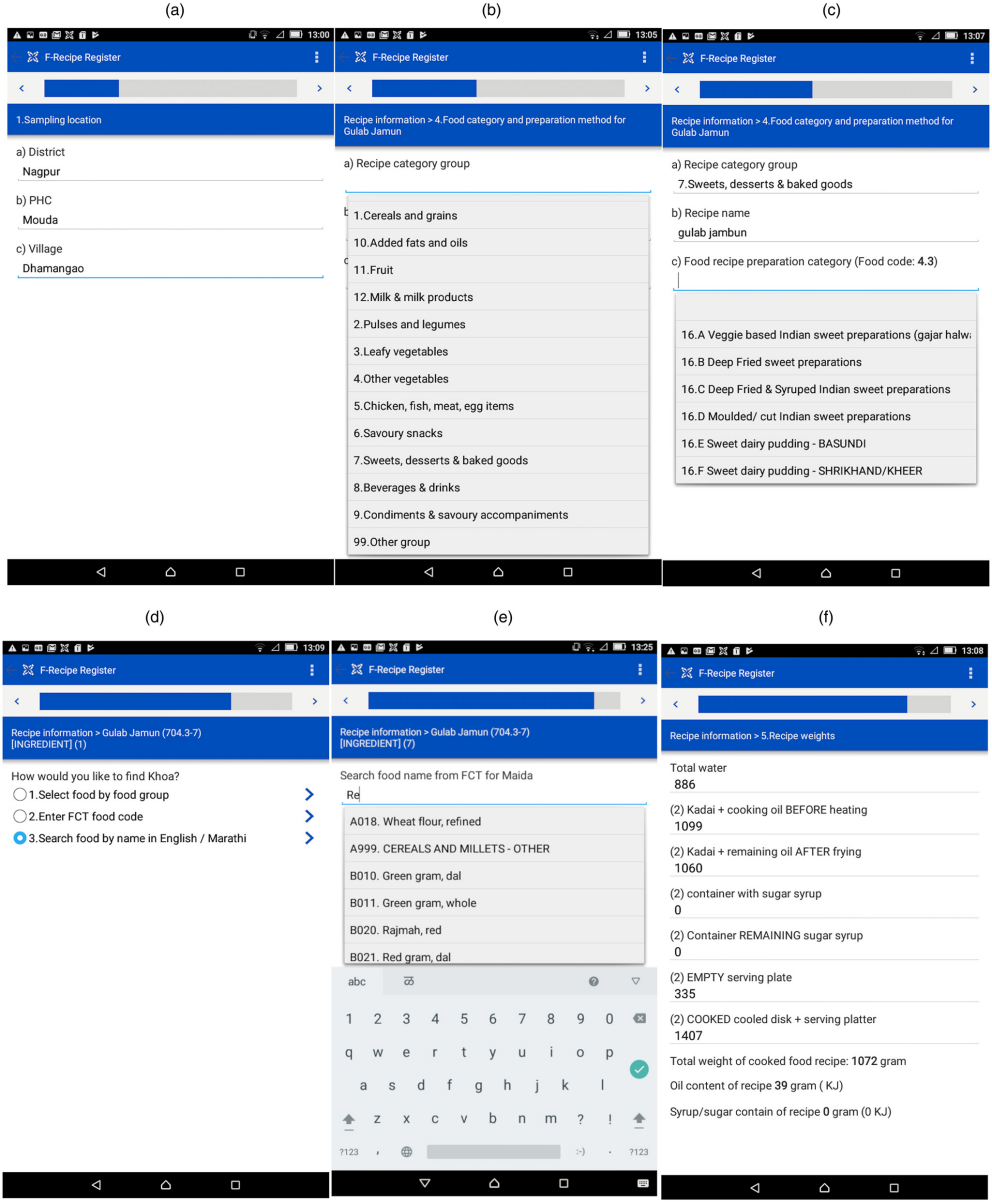
Snapshot of the user interface for the ACEC App menu: (a) selection of site location, (b) food group-specific entry of recipe, (c) selection of recipe preparation category, (d) select search option, (e) search by food name, (f) entry of total cooked recipe weight.

The user interface was bilingual in English and Marathi languages. It consisted of sequential steps or passes for data entry that appeared one at a time on the screen of the android tablet. These steps are shown in Fig. 5. The user had to choose the entry from a dropdown menu that used the backend data to fetch the information in the menu. During each data input step, the App performs a validation check for weights out of range. The snapshots (Fig. 6) display an example of an Indian sweet preparation of ‘*gulab jamun*’ where the sugar syrup was prepared separately. A progress bar displayed the number of steps completed. The snapshot of the ACEC App is shown in Fig. 6(a)–(f). When these entries are complete, the in-App summary displays the auto-calculated energy in kilojoules per 100 g for each ingredient in the recipe and the total energy for entire recipe.

### FFQ App features

**Fig. 7.**
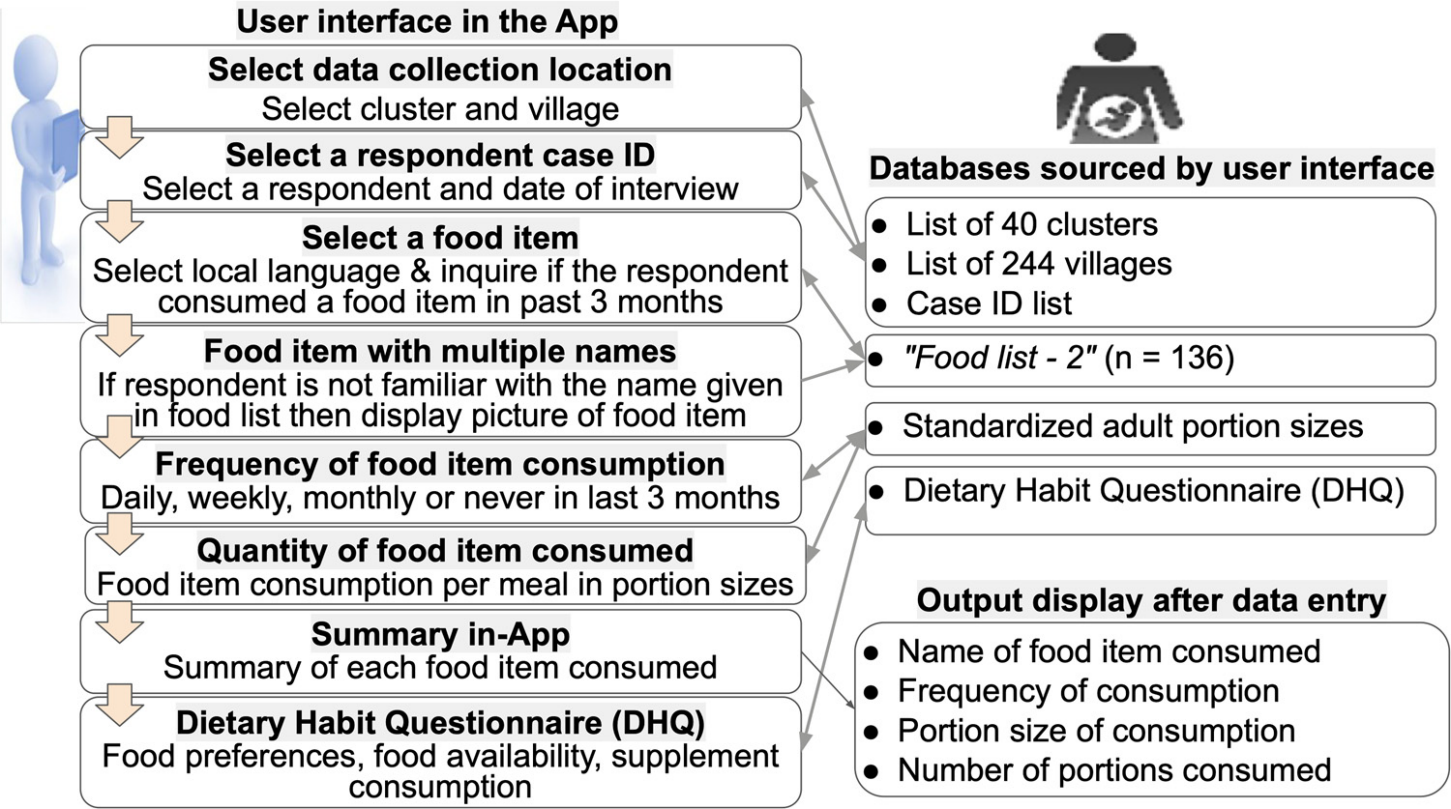
User interface and backend database of FFQ App.

**Fig. 8.**
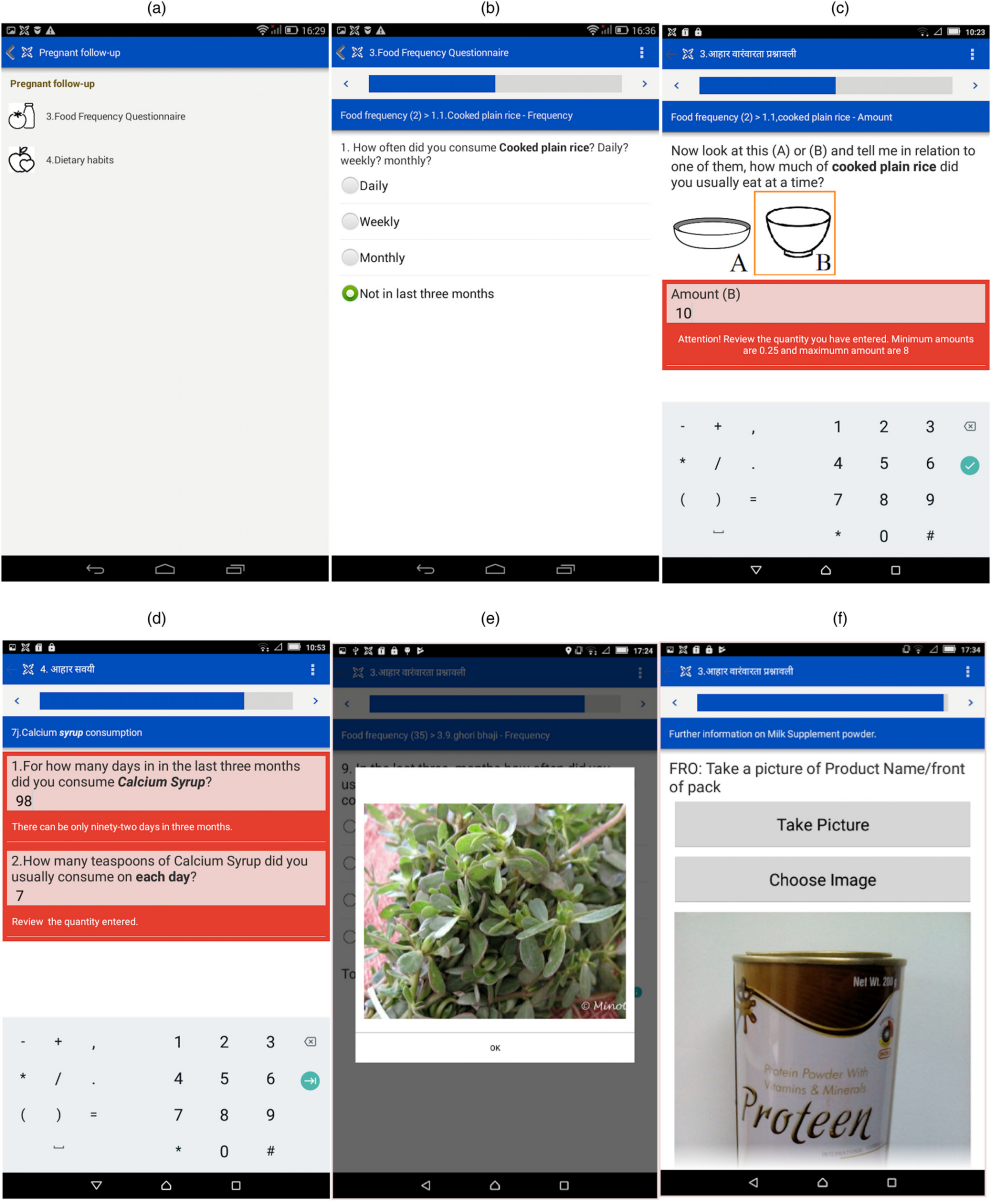
Snapshot of user interface menu: (a) selection menu: Food Frequency Questionnaire or Dietary Habits; (b) selection of food consumption frequency, (c) selection of recipe-specific portion weights, (d) validation check for numeric input, (e) image of the local vegetables, (f) data image capturing feature.

The FFQ App was also bilingual and designed with sequential steps or passes shown in Fig. 7. The App had two sections: (1) FFQ, and (2) Dietary habits. In the FFQ section, the App connected with relevant backend databases at each step to fetch the drop-down menu information. The App had a logic check that showed a red warning dialogue box if a compulsory question had been skipped (Fig. 8(d)). After the data entry of each food item, the summary output displayed the name of the food item that was consumed, the frequency of consumption, the portion size of consumption and the number of portions consumed. In the Dietary habits section, the DHQ displayed eight questions that inquire about food preference (vegetarian, non-vegetarian), food availability from *Anganwadi*, and supplement consumption (iron, folic acid) to be answered as Yes or No options. The Dietary habits section allowed for pictures of the box of supplements or wrappers of medicines to be photographed by the device camera for updating the database of food items (Fig. 8(a)–(f)).

### IDR App features

**Fig. 9.**
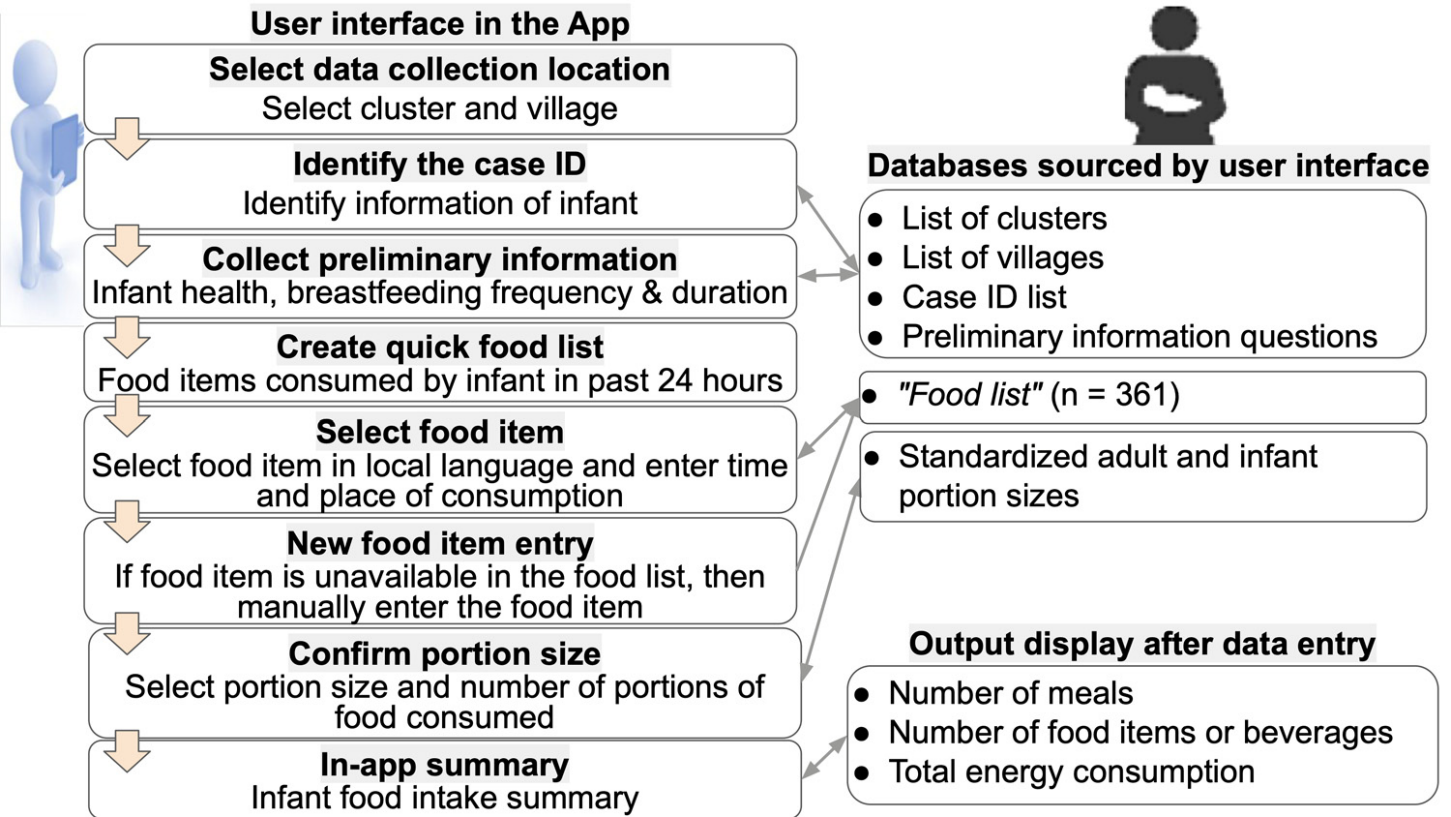
User interface and backend database of IDR App.

**Fig. 10.**
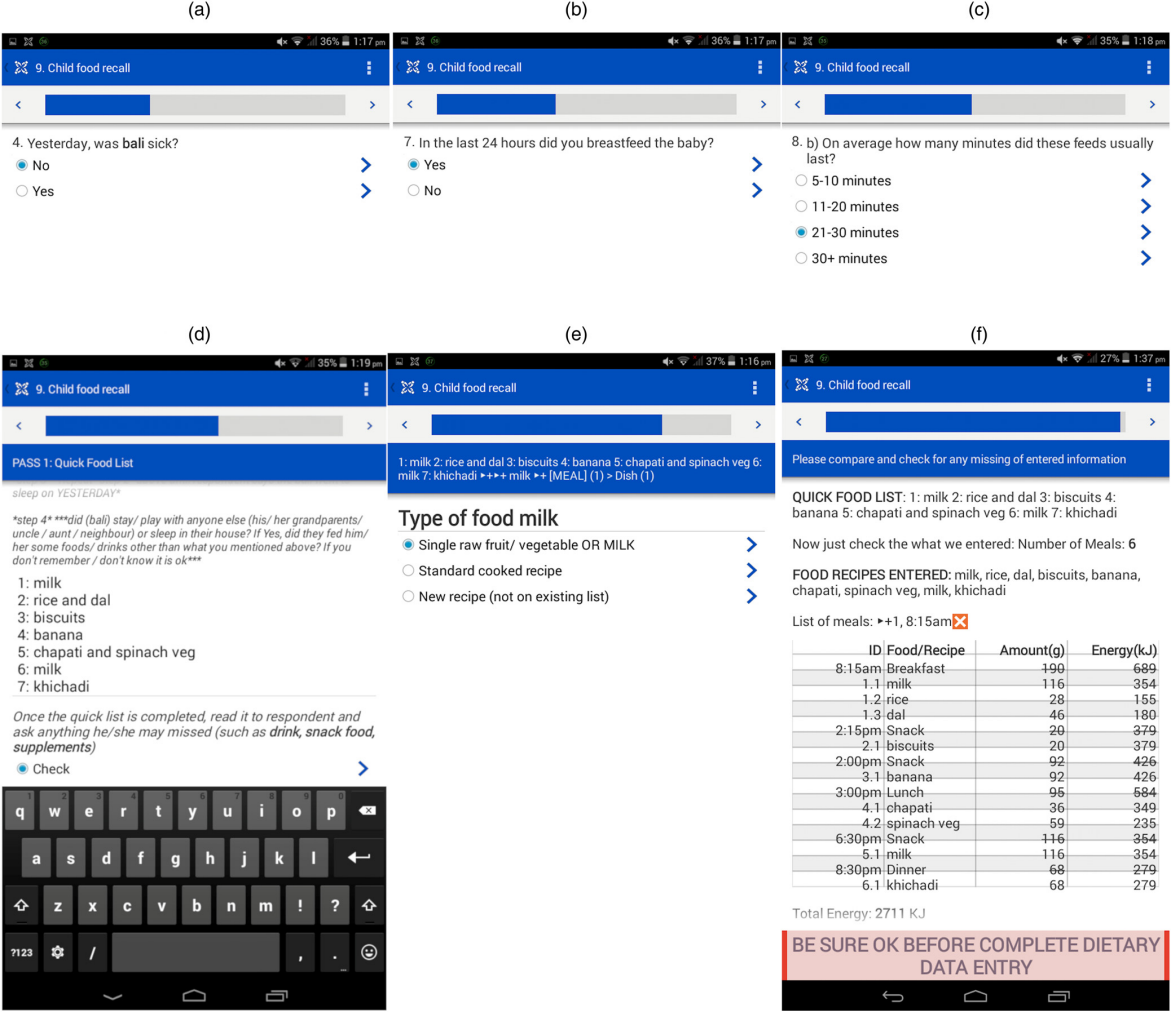
Snapshot of user interface menu: (a–c) questions about child illness and breastfeeding details, (d) quick food list, (e) real-time new food data entry, (f) summary of food intake by an infant.

The IDR App was also bilingual and consisted of user interfaces shown in Fig. 9. The App started with a display of participant cases that were due for collection of infant dietary data by an alert icon. A FRO then clicked on that specific case to open the questionnaire, which displayed a list of data entry steps or passes, Fig. 9. This App allowed for entry of a food item and its ingredients. If the food item was unavailable in the existing food list, it was appended to the existing backend food list. In the end, the App displayed a summary of the infant's food intake that consisted of the number of meals, the number of food items or beverages and the total energy consumed by the infant. Before the FRO submitted the collected data using the App, it displayed a message to verify that all data entry of food items consumed by the infant had been completed (Fig. 10(a)–(f)).

## Discussion

In this paper, we describe the development and features of three android tablet-based Apps — the ACEC, FFQ, and IDR Apps used for dietary data collection in rural pregnant women and their infants. These Apps were used by trained community ANMs to collect reliable regional dietary information for the M-SAKHI study. A comprehensive and detailed description of methods used for dietary data collection in research studies that evaluate nutrition outcomes of an intervention is needed as it informs researchers regarding the reliability of the information collected and its replicability. High-quality data are essential to study the association between diet and health. Therefore, persistent efforts are needed to improve the existing dietary assessment methods and develop innovative tools to enable and standardise them. Recently, several studies have reported the successful use of android-based technologies for dietary data collection and assessment. These studies have concluded that technology-based data collection reduces errors and improves the quality and accuracy of dietary data and its analysis.^(17–23)^ The novel element of our methodology was the ACEC App that helped to create a standardised regional generic cooked food recipe database with their ingredients, portion sizes and energy value. This food database was then used on the backend to support FFQ and IDR Apps for dietary data collection and then the nutrient assessment of the diet consumed by pregnant women and infants. Similar dietary recall software applications have been developed and used in previous studies where the food item selection was from publicly available databases or food composition tables (FCT).^(24,25)^ In contrast, in the current study, a key innovation was the preparation of the regional food list, their recipes and a portion kit that is regionally appropriate prior to collect dietary data to ensure authentic estimation of energy values of consumed food. A key factor in the development of ACEC, FFQ, and IDR Apps was that they were developed in consultation with regional experts in the field of nutrition with substantial experience in dietary assessment methodologies. The major strengths of these Apps are that they are simple and user-friendly. Data collectors can use them with basic skills in the usage of android tablets and basic knowledge of regional diets. These Apps have standard questions, allowable inputs, features of validation of erroneous or out-of-range data entries and reminders to ensure standardisation of responses and completeness of data. Ready access to databases connected via the App enables error-free selections of ingredients, their portion sizes, standardisation of cooked food weights and automated and error-free calculation of their energy values. Study investigators then upload these to the cloud for further monitoring and verification. These Apps also provide an opportunity to append the existing database with food items that were not on the food list, to begin with. The updated lists are then useful for current and future nutrition studies. The FFQ App had an option of taking pictures of food items or supplements not otherwise recognised by the data collectors. These features significantly improve the quality of the data collected compared to a traditional paper-based approach.

Other types of software applications for dietary data collection are also available for nutrition research studies. They can be linked with external electronic gadgets such as armbands, smart watches or bar code readers from capturing data.^(26)^ For example, an atlas of recipes and their portion sizes with accompanying Quick Response codes (QR codes) have been used for expedited dietary data collection in Nepal. The pictures of recipes in the atlas are shown to the respondent who identifies the recipe. The data collector scans the QR code, and information regarding the recipe ingredients and portion size is uploaded to the database of an android device.^(27)^ However, the limitations are that the atlas may not contain an exhaustive list of food items consumed in that region. Also, if the food item in the atlas contains ingredients different from those used by the respondents, then there is no opportunity to update the atlas. In our study, we could append new recipes to our existing list using the ACEC App. Recently, some apps have used artificial intelligence (AI) to automate the task of detecting and classifying a recipe using pictures of a recipe.^(20,21)^ While research efforts are underway for using AI for dietary data collection, accurately identifying ingredients in a multi-ingredient recipe remains difficult. Indian food recipes are multi-ingredient and regionally diverse, even within a state. To overcome this challenge, we invited women from different households to cook the recipes in the presence of nutritionists to create the generic recipe database using the ACEC App. AI can help to create a generic recipe, but information regarding the different ingredients used would need to be collected from the participants. Thus, the ACEC App can be used across other regions to create regional cooked food recipe databases, as commercially available nutrition Apps or software programmes do not have information on many Indian regional cooked food recipes.

Thus, apart from the advantages of automation, the ACEC App also offers the potential to gather dietary data from a wide range of culturally diverse cuisines worldwide due to its provision for entering new recipes. However, it is important to note that this hinges on the inclusion of the ingredients of these new recipes into the backend database. Likewise, the FFQ App can be employed for collecting dietary data from various countries, necessitating updates to the food list in the backend database to encompass new food items. The IDR App also has applicability to collect infant dietary data from different countries, contingent upon the utilisation of a backend database containing new recipe information. The scalability of the Apps makes them amenable for efficient dietary data collection from larger populations. Also, the learning curve associated with using the Apps is gradual, requiring less time to train the data collectors.

These digital tools have proven to be suitable for large-scale studies that are technically complex but have low financial resources, high running costs and a small-time window to conduct field research. Mobile software applications that could be deployed on a large scale for a 24 h recall have reduced the project cost by up to 45 % compared to a traditional pen and paper-based approach.^(28)^ These applications allow investigators to expedite data collection and seamlessly integrate it with an internet database to facilitate data processing and analysis.^(29)^ High-quality data can thus be collected from several geographical locations and consolidated into a central database. This will foster collaboration, information sharing and regular updating regional food recipes in India.^(30)^ The ACEC, FFQ, and IDR Apps developed in this study are important tools to achieve this goal.

A limitation of this study was that the ACEC App's database solely encompasses ingredients from the central region of India. Additional work is needed to augment the database with ingredient data from different locations to enable the input of recipe information from diverse areas. Both the ACEC and IDR Apps were designed for energy calculations exclusively. Expanding these calculations to encompass other nutritional values falls outside the scope of the current study. The Apps currently offer support for only two languages: English and Marathi. Finally, an inherent challenge tied to accessing the Apps at zero cost revolves around intellectual property rights linked to the developer platform, which might potentially limit access to App usage without a paid subscription.

## Conclusions

In the M-SAKHI study, innovative android-based Apps, namely ACEC, FFQ, and IDR were developed to collect high-quality dietary data of rural pregnant women and infants from villages in Maharashtra in central India. The ACEC App was used to create a region-specific food database. In contrast, the FFQ and IDR Apps were used to collect dietary data to evaluate the dietary diversity and nutrient values of foods that rural pregnant women and their infants consumed. These Apps are user-friendly and have several built-in features that ensure high-quality data are captured on the field. Furthermore, these Apps are an especially promising alternative for collecting dietary data in a low-resource setting to empower researchers and policymakers with actionable data.
